# Limited Stress Surface Model for Bending and Torsion Fatigue Loading with the Mean Load Value

**DOI:** 10.3390/ma14227023

**Published:** 2021-11-19

**Authors:** Roland Pawliczek, Dariusz Rozumek

**Affiliations:** Department of Mechanics and Machine Design, Faculty of Mechanical Engineering, Opole University of Technology, 45-271 Opole, Poland; d.rozumek@po.edu.pl

**Keywords:** mean stress, fatigue life, bending load, torsional load

## Abstract

In this study, a linear model of the transformation of the stress amplitude due to the mean value was used. The coefficient of the material sensitivity to cycle asymmetry with consideration of the dependence of this coefficient on the number of fatigue loading cycles is also used. A three-parameter surface model of limited stresses is proposed in this paper. The model is verified using the results of fatigue tests for cyclic bending and torsion under mean loads. The tests are performed for two types of alloy steels—S355J0 and S355J2G1W. Comparison of the allowable stress amplitudes obtained experimentally with those predicted using the proposed model shows errors of no more than 18%, with the area of the surface with the largest error being relatively small.

## 1. Introduction

Structures and elements of machines can be subjected to time-varying loads, which cause fatigue processes in the materials. These loads are most often of a complex nature. Additionally, the mean load appears resulting from the self-weight of the structure or initial loads related to the functions fulfilled by the structure. The asymmetry of the fatigue load significantly influences the change of the allowable amplitudes; therefore, an understanding of the mutual relations between the average load and the maximum variable load is important when determining the durability of materials operating in such conditions. In the literature, mathematical models of the influence of the mean load on the fatigue life of construction materials are usually based on the basic strength properties of the material, such as the strength or yield point. In engineering practice, these properties are convenient to use because they do not require complex calculations or separate tests to determine the model parameters (material properties).

The commonly used transformation relationships based on changes in the mean stress values from zero are the Goodman, Gerber, and Marin models, which assess the mechanical properties of materials using static tensile tests [1,2,3,4]. In these cases, reservations are raised by the reference to the static properties of the material, which are not necessarily adequate for the fatigue phenomenon. On the other hand, the Morrow and Kliman models use material constants that characterize the fatigue properties of the material [5,6]. These are the parameters describing the Manson–Coffin–Basquin fatigue strain characteristics, namely the fatigue strength exponent and exponent of fatigue plastic strain. Another very popular model was presented by Smith, Watson, and Topper (SWT) [7]. This model is usually used in strain based fatigue analysis by combining the SWT parameter with the strain–life equation proposed by Manson [8] multiplied by the Basquin power law relation [9]. With the assumption that the plastic part of the strain amplitude is small and can be neglected during the fatigue life assessment, which is a typical practice in high-cycle fatigue, this model can only be expressed in terms of stress. A generalization of the SWT model, taking into account the mean value of the load, is the Walker model [10], where in special cases it is synonymous with the SWT model.

As mentioned, the described models are widely used and have been repeatedly verified by fatigue tests under the mean load. The tests can cover various types of materials (steels, aluminum alloys) and loads (uniaxial and multiaxial, cyclic or random).

The well-known Goodman and Gerber relationships were verified by testing alloy steel under random loading conditions, which were treated as the most general conditions [11]. While investigating different methods of assessing the mean value in the random waveform of loading, it was shown that irrespective of the method used for counting the cycles, the Goodman transform relation gave satisfactory results for fatigue life estimation.

Additionally, Pawliczek and Kluger [12] showed that for random loads with mean values, the load irregularity factor is an important parameter. When increasing the value of this coefficient, differences appear in the calculation of the fatigue damage accumulation factor for each transformation model due to the mean value. In this case, the Goodman model proved to be the most sensitive, with the differences in calculations being significant.

It is also pointed out in the literature that the limitation of the dependence of the Goodman and Gerber model is significant at the fatigue limit level. Gasiak and Pawliczek [13] and Kluger et al. [14] showed that the parameters of computational models considering the mean value change with the number of cycles. Verification of the computational models for so-called life-dependent material parameters was performed for specimens made of S355 alloy steel [13,14] and 7075-T651 aluminum alloy [14]. Taking these effects into account, a more accurate estimation of the fatigue life of the materials was achieved.

For complex multiaxial loading, the use of critical plane-based computational models is suggested to describe fatigue phenomena, whereby the stress state is analyzed at a plane considered critical for the material in terms of fatigue stress. Both normal and tangential stresses in this plane are considered here as criterion quantities [15,16]. In addition, these models consider a set of parameters describing the effects of load multiaxiality. The so-called energy models are also used in the description of fatigue tests. These models relate to the energy accumulated in the material during successive loading cycles, as well as stresses and strains in the material, and refer to a critical level of strain energy. Comparative analysis [16] has shown better performance of these models compared to critical-plane-based models. Several relationships for considering the average load for both the energy models and those based on the critical plane (e.g., McDiarmid, Papadopoulos, Smith–Watson–Topper, Łagoda and Macha) were verified for studies of steel alloy S355J0 and 30NCD16 and aluminum alloy 2017A-T4 [15,16]. For all analyzed types of loads, with the use of the stress–strain energy parameter, which takes account the effects of the mean stress value, the results were close to the experimental results. The tests were conducted under conditions of plane bending, torsion and with two combinations of proportional bending with torsion to assess the contribution of the mean stress value. An interesting proposal to consider the mean value for torsional loads was proposed by Bohm and Nieslony [17]. In a linear model used to calculate the equivalent stress amplitude, they proposed a correction factor based on interpolation of the stress amplitude for any value of the stress ratio −1 < R < 0 based only on fatigue characteristics obtained under loading conditions, for which R = −1 and R = 0. The life estimation algorithm was verified for drilled specimens made of three aluminum alloys. For tests under torsional conditions involving the mean value, good agreement was obtained for the life estimation based on the baseline fatigue characteristics of the material at the zero mean value.

Pawliczek and Łagoda pointed to the problem of changes in material properties caused by mean loads [18]. On the basis of studies available in the literature, they performed simulation calculations, indicating that for materials particularly sensitive to cycle asymmetry, changes in the strain–stress curve parameters result in large errors relating to fatigue damage accumulation if these changes are not taken into account. This is especially true for low-cycle loading.

Dowling [19] and Dowling et al. [20] conducted a comparison between the well-known Smith–Watson–Topper (SWT) model and the extended model proposed by Walker for mean value loads. Although the SWT model gave good results for fatigue life estimation, Walker’s model for low cycle loads proved to be better. The only disadvantage of the Walker model was the difficulty in determining one of the equation parameters in the experimental method. A set of 18 different materials [19,20] from both steel and aluminum alloy groups were analyzed. Ince and Glinka [21] indicated that in general, the SWT model expressed in stress equations reduces its application to high-cycle loading. Extending the SWT model to the range of low-cycle loading, the authors proposed the introduction of equivalent strains. The proposed model considers elastic and plastic strains and introduces a correction due to the mean value of the load. The proposed mean stress correction model was found to be better than the SWT model for Incoloy 901 superalloy and ASTM A723 steel, while both models provided equally good correlations with experimental data for 7075-T561 aluminum alloy and 1045 HRC 55 steel.

With respect to computational models based on stress–life (σ-N) or strain–life (ε-N) curves, Kwofie and Chandler [22] proposed a correction that allows the use of the SWT model for materials that show an increase in fatigue life when the average load is increased (e.g., copper). The so-called mean stress sensitivity index can be determined from experimental data. The effect of the increase in durability with an increase in the mean load itself is explained by the natural tendency of the mean tensile load to enlarge a fatigue crack. When the average load is very high, this could counteract the effect of the decreasing plastic strain amplitude. The second element is the effect of cyclic creep, which usually occurs for this type of material.

In general, it can be concluded that it is not possible to indicate one universal calculation method due to the mean load, as both these factors affect the efficiency of calculations. Numerous effects and parameters that are indicated as important elements for estimation relate to material properties and loading conditions. Simple computational models (e.g., Goodman and Gerber models) do not guarantee good results for durability estimation, which is important from the point of view of structural safety. A number of algorithms indicate the necessity of basing such estimations on the results of experimental studies. The purpose of this study is to learn the behavior of specimens made of S355J0 and S355J2G1W steels under conditions of cyclic bending and torsional loading with the participation of various mean values. In this paper, using a model of the transformation of stress amplitudes due to the mean value, a general three-parameter limiting stress surface is defined in the form of a relationship between the stress amplitude, mean stress value and the number of fatigue cycles.

## 2. Transformation Model for the Mean Load Value in Relation to the Number of Fatigue Cycles

The previously presented calculation models contain material properties that are constant values. Meanwhile, some research results indicate that the fatigue properties of the material may change with the number of cycles, and it is postulated that this effect should be taken into account in calculation algorithms [14,16,23,24]. Some proposals extend the applicability of the Goodman, Gerber, and other mentioned models in the range of low-cycle fatigue by taking into account the current number of failure cycles in transformation models [25,26].

**Figure 1:**
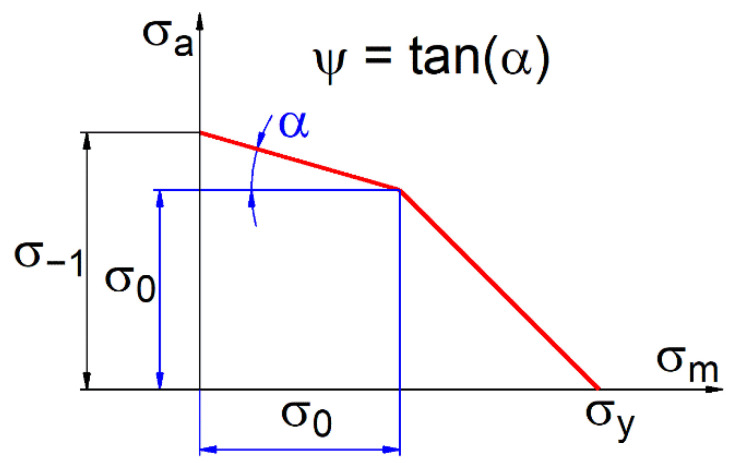
Interpretation of the cycle asymmetry sensitivity ratio based on the two-parametric characteristic σ_a_ = f(σ_m_).

In this paper, the influence of the mean load value on the limit amplitude value can be described by the cycle asymmetry sensitivity ratio ψ, which is defined according to the scheme of the two-parametric characteristic σ_a_ = f(σ_m_). A simplified bilinear model of this characteristic is presented in Figure 1.

The cycle asymmetry sensitivity ratio ψ can be expressed as:

(1) $$\psi \;=\;\frac{\sigma_{-1}\;-\;\sigma_{0}}{\sigma_{0}},$$

where σ_−1_ is the stress amplitude for fatigue tests results under purely alternating loads (R = −1), σ_0_ is the stress amplitude for fatigue tests results under purely pulsating loads (R = 0), and R is the stress ratio R = σ_min_/σ_max_.

**Figure 2:**
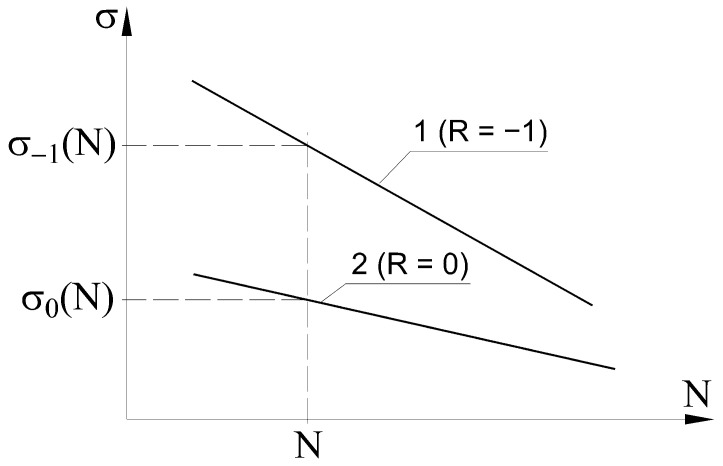
Determining the dependence of the material sensitivity coefficient to cycle asymmetry on the number of cycles.

It is assumed that the factor ψ may change with the number of cycles N. Figure 2 shows the scheme used for determining the value of factor ψ depending on the number of N cycles.

If the results of fatigue tests for purely alternating loads (R = −1) are described with line 1 in Figure 2, and for pulsating loads with line 2 in Figure 2, the material’s sensitivity coefficient to cycle asymmetry for the life of N cycles can be calculated as:

(2) $$\psi (N)\;=\;\frac{\sigma_{-1}(N)\;-\;\sigma_{0}(N)}{\sigma_{0}(N)},$$

where σ_−1_(N) is the stress amplitude at the N cycle life level for loads with the cycle asymmetry factor R = –1 and σ_0_(N) is the stress amplitude at the durability level of N cycles for loads with the cycle asymmetry factor R = 0.

Equation (2) is explained in [27] for normal and shear stresses with these formulas.

Based on the fatigue characteristics for the tests at R = −1 and R = 0, it is possible to determine changes in the material sensitivity coefficient to cycle asymmetry.

It is easy to show that if the slope is the same for straight lines 1 and 2 in Figure 2, the material’s sensitivity factor to cycle asymmetry is constant over the entire range of fatigue life. If the lines shown in Figure 1 clearly differ in their slope, then the value of the coefficient ψ changes with the number of fatigue cycles.

The changes of the ψ factor with the number of cycles N can be determined experimentally using the equation:

(3) $$\psi (N)\;=\;\eta \cdot N^{\lambda },$$

where η, λ are parameters, the values of which are determined on the basis of fatigue tests results under purely alternating (R = −1) and purely pulsating (R = 0) loads.

Taking the logarithm of Equation (3), the following expression is obtained:

(4) logψ = logη + λ⋅logN.

**Figure 3:**
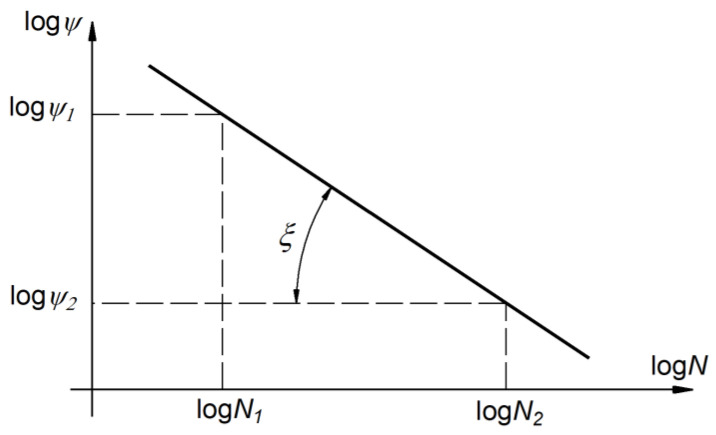
Material cycle asymmetry sensitivity factor in a bilogarithmic coordinate system.

The relationship (4) describes the equation of a straight line in a bilogarithmic coordinates system (Figure 3). The slope λ in Equation (4) is related to the angle ξ by the following equation:

(5) $$\lambda \;=\;\tan\xi \;=\;\frac{\log\psi_{1}\;-\;\log\psi_{2}}{\log N_{1}\;-\;\log N_{2}}.$$

The values of the cycle stress amplitude for purely alternating and purely pulsating loads can be determined from the equations describing the results of fatigue tests.

For purely alternating loads:

(6) $$\log N\;=\;B_{w}\;+\;A_{w}\cdot \log\sigma_{-1}\;\;\;\;\;\Rightarrow \;\;\;\;\;\sigma_{-1}\;=\;{\left(\frac{N}{10^{B_{w}}}\right)}^{\frac{1}{A_{w}}},$$

For purely pulsating loads:

(7) $$\log N\;=\;B_{j}\;+\;A_{j}\cdot \log\sigma_{0}\;\;\;\;\;\Rightarrow \;\;\;\;\;\sigma_{0}\;=\;{\left(\frac{N}{10^{B_{j}}}\right)}^{\frac{1}{A_{j}}},$$

Considering Equations (6) and (7), Equation (2) takes the following form:

(8) $$\psi (N)\;=\;N^{a}\cdot 10^{b}-1,$$

where $a\;=\;\frac{1}{A_{w}}\;-\;\frac{1}{A_{j}}$; $b\;=\;\frac{B_{j}}{A_{j}}\;-\;\frac{B_{w}}{A_{w}}$,

Formula (5) for the calculation of the coefficient λ takes the following form:

(9) $$\lambda \;=\;\frac{\log\left(N_{{}_{1}}^{a}\cdot 10^{b}-1\right)\;-\;\log\left(N_{{}_{2}}^{a}\cdot 10^{b}-1\right)}{\log N_{1}\;-\;\log N_{2}},$$

According to Equation (3) and considering Equation (8), the coefficient η can be determined as:

(10) $$\eta \;=\;\frac{\psi (N)}{N^{\lambda }}\;=\;\frac{N_{{}_{1}}^{a}\cdot 10^{b}\;-\;1}{N_{1}^{\lambda }}\;=\;\frac{N_{{}_{2}}^{a}\cdot 10^{b}\;-\;1}{N_{2}^{\lambda }}.$$

Equations (9) and (10) allow us to determine the values of the parameters λ and η in Equation (3) describing the function of the change in the value of the coefficient of material sensitivity to cycle asymmetry with the number of cycles N.

## 3. Limiting Stress Amplitude Surface Model

**Figure 4:**
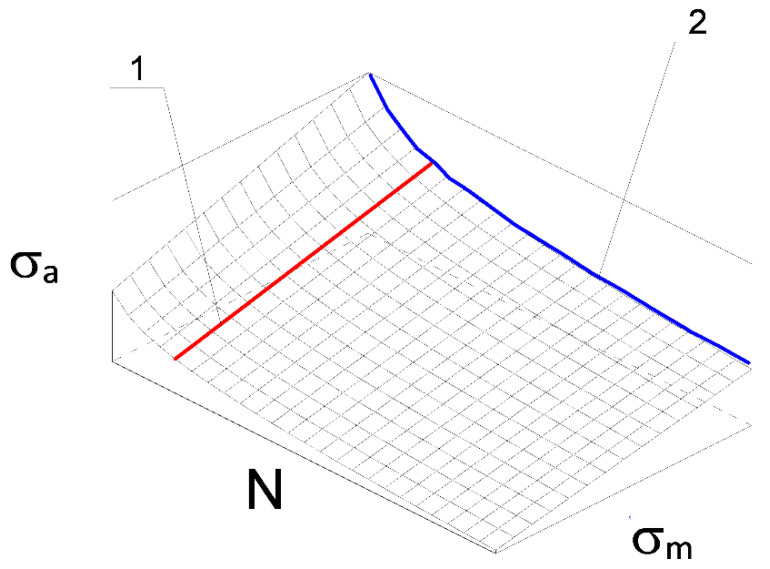
Limiting stress amplitude surface σ_a_ = f(N, σ_m_): 1—function describing influence of mean stress value on permissible stress amplitudes σ_a_ = f(σ_m_), 2—fatigue characteristics of material σ_a_ = f(N).

During the design of machine elements or structures due to the phenomenon of fatigue, loading conditions are determined for the expected life of the elements. In the case of cyclic loads with the contribution of the mean stress, the limiting stress surface is represented in the σ_a_-σ_m_-N coordinate system as shown in Figure 4.

The mathematical description of the surface is a function f(σ_a_, N, σ_m_) that relates the stress amplitude, mean value, and number of failure cycles. The search for a surface model based on fatigue test results is a complex and complicated issue [28]. The surface shown in Figure 4 carries information about the mutual relations of stress amplitude σ_a_, mean stress value σ_m_ (curve 1 in Figure 4), stress amplitude σ_a_, and number of failure cycles N (curve 2 in Figure 4). Assuming a certain function σ_a_ = f(σ_m_) to describe curve 1 and σ_a_ = f(N) to describe curve 2, it is possible to determine the mathematical form of the limiting stress surface. The accuracy of the mathematical model of the surface depends on the accuracy of the adopted mathematical models describing curves 1 and 2.

Taking into account the coefficient of the material’s sensitivity to cycle asymmetry and its interpretation presented in Figure 1, the relationship between the allowable stress amplitude and the mean stress value can be described by the following formula:

(11) $$\sigma_{a}\;=\;\sigma_{-1}\;-\;\psi \sigma_{m},$$

where σ_a_, σ_m_ are the amplitude and mean value of the asymmetric load (R ≠ −1) and σ_−1_ is the stress amplitude of the symmetric (purely alternating) load (R = −1) equivalent in terms of fatigue to the asymmetric load.

Assuming that the effects of the mean value of the material are different for different numbers of cycles N applied to the material and using Equation (3) to describe the change the coefficient of material sensitivity to cycle asymmetry with the number of cycles to failure, Equation (11) can be written in the following form:

(12) $$\sigma_{a}\;=\;\sigma_{-1}(N)\;-\;\psi (N)\cdot \sigma_{m}\;=\;\sigma_{-1}(N)\;-\;\eta \cdot N^{\lambda }\cdot \sigma_{m},$$

Experimental fatigue characteristics of the material for purely alternating loads (R = −1, curve 2 in Figure 4) are described by the following equation:

(13) $${\left(\sigma_{-1}(N)\right)}^{m}\cdot N\;=\;\sigma_{f}^{m}\cdot N_{0},$$

where m is the slope of the Wöhler curve, σ_f_ is the fatigue limit, and N_0_ is the limit (edge) number of load cycles.

Determining the stress amplitude σ_−1_(N) from Equation (13) and inserting it into relation (12), one obtains the limiting stress amplitude surface equation in the following form:

(14) $$\sigma_{a}(\sigma_{m},N)\;=\;{\left(\frac{\sigma_{f}^{m}\cdot N_{0}}{N}\right)}^{\frac{1}{m}}\;-\;\eta \cdot N^{\lambda }\cdot \sigma_{m}.$$

Equation (14) represents a mathematical model of the limiting stress amplitude surface determined from fatigue test results for purely alternating and purely pulsating loads, assuming that the effect of the mean value on the stress amplitude is a linear function.

## 4. Fatigue Tests

The subjects of the study are S355J2G1W steel (European Standard EN 10155:1993, Low-alloy rust-resistant steel, The European Committee for Standardization) and S355J0 steel (European Standard EN 10025:2019, Hot rolled products of structural steels, The European Committee for Standardization) [12,27,29]. The strength properties are presented in Table 1.

**Figure 5:**
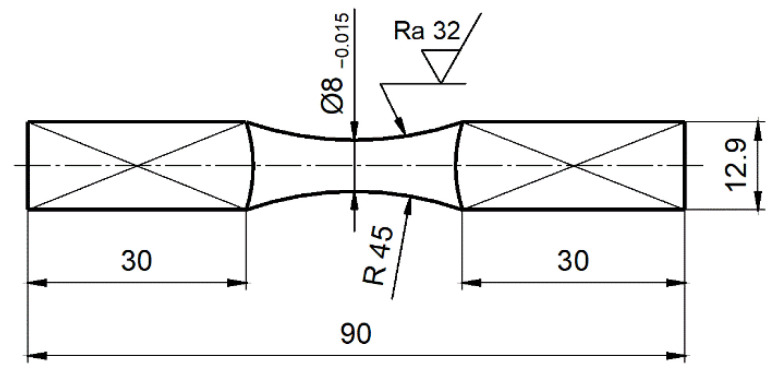
Shapes and dimensions of specimens (dimensions shown in mm).

Figure 5 shows the shape and dimensions of the specimen used in the tests.

The tests were carried out on the MZGS–100 fatigue test stand (Opole University of Technology, Opole, Poland) [30]. The stand allows testing for fatigue bending or torsion and additionally proportional bending with torsion (not applied in this research). The tests of cyclic bending and torsion were performed within the low and high range numbers of cycles where the moment amplitude was controlled. The loads present sinusoidal waveforms under a frequency range of 25–29 Hz. The nominal stress amplitude and nominal mean stress value were used in calculations.

The results of fatigue tests were approximated by the following regression equation:

(15) $$\log N\;=\;B\;+\;A\cdot \log\sigma_{a}$$

where σ*_a_* is the bending stress amplitude (for torsion accordingly τ*_a_*), *N* is the number of cycles until failure, and *A* and *B* are the parameters of the regression equation.

The parameters in Equations (3), (14) and (15) were determined based on fatigue test data from Pawliczek and Rozumek [27,29]. Fatigue tests were performed under cyclic bending and cyclic torsion with the mean value of the load at fixed stress ratios R = −1, R = −0.5 and R = 0 for S355J0 steel and for fixed mean stress values σ_m_ (τ_m_) 0, 75, 150 and 225 for S355J2G1W steel. For each load level, the tests were carried out with a minimum of 3 specimens. A set of loading conditions for fatigue tests is presented in Table 2.

Table 3 shows the values of coefficients A and B of regression Equation (15) for bending and torsion and the stress ratios R = −1 and R = 0, respectively.

Analyzing the parameters contained in Table 3, it can be concluded that the values of the A coefficient for bending and the presented materials are different in all cases. During torsion, for R = −1, the values of the coefficient A show smaller differences than for R = 0, where greater differences between their values are noticed.

**Figure 6:**
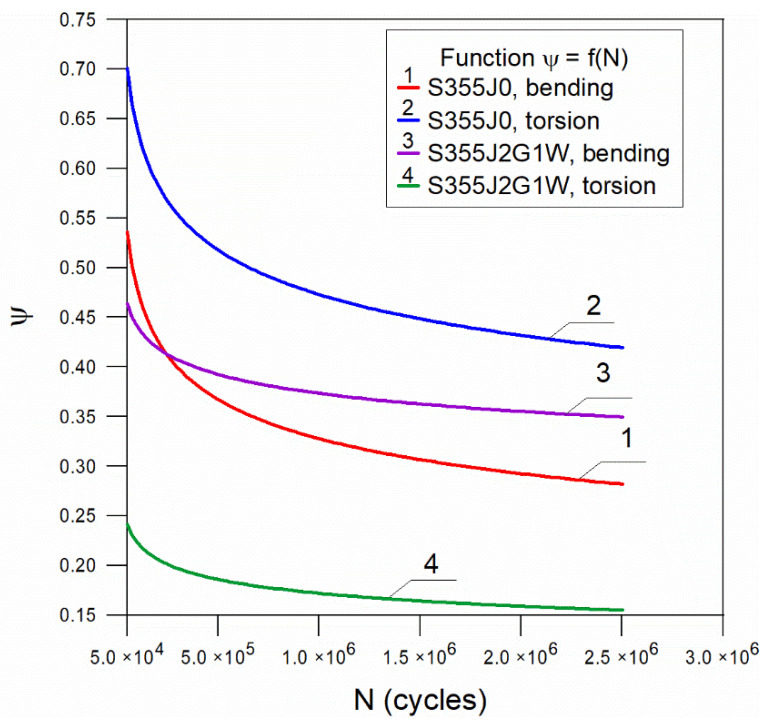
A graph of the function ψ = f(N) for tested steels.

Based on the data in Table 3, the coefficients η and λ of Equation (3) were determined. The results of the calculations are shown in Table 4 and Figure 6 shows the functions for the tested steels.

The values of η and λ coefficients for bending and torsion show similar effects of the mean stress value on the allowable stress amplitudes for both steels. Figure 6 also indicates that for the durability range of 5 × 10^4^ to about 7.5 × 10^5^ cycles, the value of the coefficient ψ decreases significantly, while for the high-cycle durability range, the value of the material’s sensitivity factor to cycle asymmetry changes slightly.

In the case of S355J0 steel bending, this relationship was described by the function ψ(N) = 3.16N^−0.164^. There is a visible decrease in the value of the factor in terms of durability at N = (5 × 10^4^–7.5 × 10^5^) cycles. In the case of torsion, the function has the form ψ(N) = 2.89N^−0.131^, while the nature of the curve is similar to that for bending.

In a similar way for S355J2G1W steel, we have ψ(N) = 1.01N^−0.072^ functions for bending and ψ(N) = 0.82N^−0.113^ for torsion, respectively. The nature of the functions is similar to that of S355J0 steel. However, it can be noted that the range of changes in ψ for the maximum value at N = 5 × 10^4^ and minimum value at N = 2.5 × 10^6^ for S355J0 steel is about 2 times larger than for the second material. This suggests a greater sensitivity of the material to cycle asymmetry, especially in the low cycle load range.

## 5. Analysis of the Limiting Stress Amplitude Surface Model

This paper presents a mathematical model of the surface description of allowable stress amplitudes depending on the value of the mean load and the number of cycles to failure of the material. It was assumed that the influence of the mean load value on the allowable stress amplitudes depends on the material’s sensitivity to cycle asymmetry. A function was proposed to describe the change in the value of the material’s sensitivity coefficient to cycle asymmetry, and its parameters were determined from fatigue tests of specimens under purely alternating (R = −1) and purely pulsating (R = 0) loading conditions.

The analysis of the limiting stress amplitude surface model was based on the data shown in Table 3 and Table 4. The experimental surfaces results for tests with stress ratios R = −1 and 0 are presented in [27,29]. The experimental surfaces of the limiting stress amplitudes were determined using Kriging multiparameter approximation [31], then the mathematical formula based on Equation (14) for the surfaces describing the different loading cases for both steels was determined.

**Figure 7:**
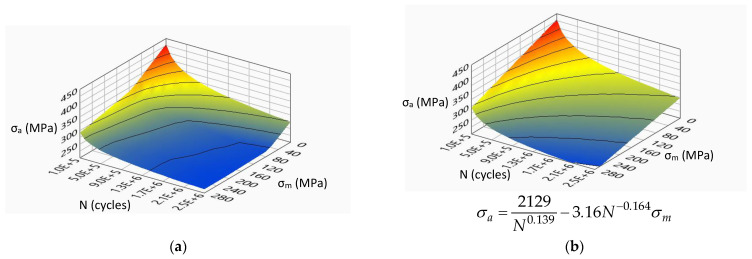
Limiting stress amplitude surface for S355J0 steel under bending load: (**a**) experimental model; (**b**) mathematical model.

**Figure 8:**
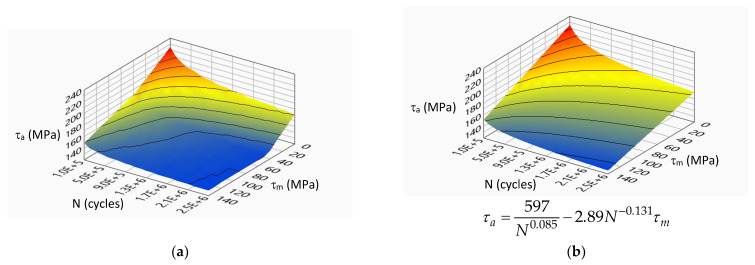
Limiting stress amplitude surface for S355J0 steel under torsion load: (**a**) experimental model; (**b**) mathematical model.

**Figure 9:**
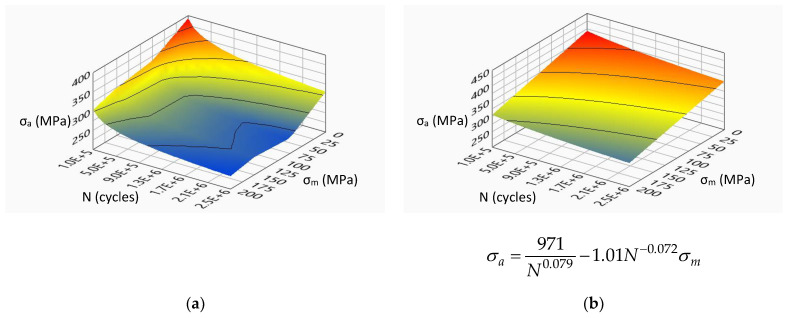
Limiting stress amplitude surface for S355J2G1W steel under bending load: (**a**) experimental model; (**b**) mathematical model.

**Figure 10:**
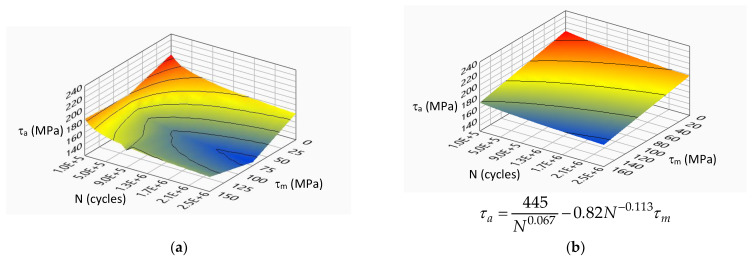
Limiting stress amplitude surface for S355J2G1W steel under torsion load: (**a**) experimental model; (**b**) mathematical model.

Figure 7, Figure 8, Figure 9 and Figure 10 show the experimental surfaces and their mathematical expressions for analyzed steels and load cases, respectively.

To determine the effectiveness of the results from predicting allowable stress amplitudes σ_a_ using the proposed model, the relative error of the stress amplitudes calculated from the theoretical model versus the experimental results was determined:

(16) $$\delta \;=\;\frac{\sigma_{a(model)}\;-\;\sigma_{a(exp)}}{\sigma_{a(exp)}}\;100\%.$$

**Figure 11:**
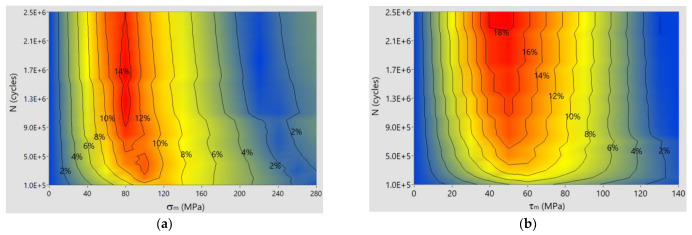
Relative error of calculated stress amplitudes versus experimental results for bending and torsion of S355J0 steel: (**a**) bending: (**b**) torsion.

Figure 11 shows the distribution of relative error as a function of mean stress and number of cycles N for specimens made of S355J0 steel.

The maximum error for bending is 14% at σ_m_ = 80 MPa and life N > 1 × 10^6^ cycles. For torsion, the error is 16% for τ_m_ = 40 ÷ 60 MPa and life N > 1 × 10^6^ cycles.

**Figure 12:**
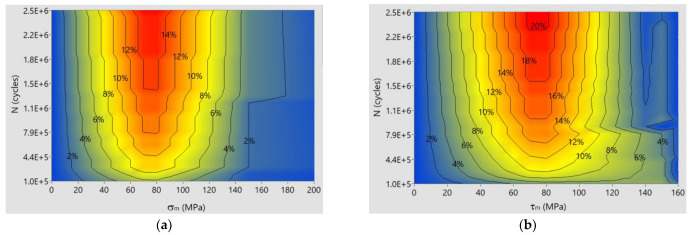
Relative error levels of calculated stress amplitudes versus experimental results for bending and torsion of S355J2G1W steel: (**a**) bending; (**b**) torsion.

Figure 12 shows the relative error areas of the calculation results versus the experimental results for bending and torsion of S355J2G1W steel specimens.

The maximum error for bending is 14% at σ_m_ = 75 MPa and life N > 1.5 × 10^6^ cycles. For torsion, the maximum error is 18% for τ_m_ = 60 ÷ 90 MPa and life N > 1.5 × 10^6^ cycles. For durability N > 2.3 × 10^6^ and τ_m_ = 75 MPa, the error reaches 20%, although this error covers an insignificant region of the plane of limit stresses.

## 6. Conclusions

On the basis of the obtained results of experimental research and the conducted analyses, the following conclusions were formulated:

1. The effect of the mean load value on the allowable stress amplitudes was taken into account by means of a linear relationship, in which the variation of the material’s sensitivity coefficient to cycle asymmetry with the number of cycles to failure was taken into account;
2. A mathematical model of limiting stress amplitude surfaces was determined, which was verified by the results of fatigue tests for S355J0 and S355J2G1W steel specimens subjected to cyclic bending and torsional loading with different values of the mean load. For both S355J0 and S355J2GW1 steels in bending, a maximum error of 14% appeared for fatigue life *N* values greater than 1 × 10^6^ cycles and a mean stress value of about 80 MPa. For torsion, similar trends were observed for both materials, with an error of 18%. Compared to bending for torsional loading, the estimation error of more than 14% covered a wider range of the average load in the range of about 40–90 MPa;
3. For both tested materials, it was observed that the greatest error in estimating allowable stress amplitudes occurred for a life of N = 1 × 10^6^ cycles, which may have been due to the largest scatter of experimental tests for loads in the high cycle fatigue range;
4. The proposed model proved to be effective in estimating the maximum stress amplitude values for low-cycle fatigue over a wide range of mean loads, where the estimation error was less than 10%.

The algorithm proposed in this paper for determining the values of permissible stress amplitudes provided consistent results with those of the experimental tests. The model can be applied to components subjected to bending as well as torsional loads. This requires that the characteristics for purely alternating and purely pulsating loads are appropriate for the type of loading.

## Figures and Tables

**Table 1:** Mechanical properties of tested materials.

Material	Yield Strength σ*_Y_* (MPa)	Ultimate Strength σ*_U_* (MPa)	Young Modulus E (GPa)	Poisson’s Ratio v
S355J2G1W	418	566	215	0.29
S355J0	357	535	210	0.30

**Table 2:** A set of loading conditions for fatigue tests of specimens.

Material	Bending		Torsion	
	σ*_m_* (MPa)	σ*_a_* (MPa)	τ*_m_* (MPa)	τ*_a_* (MPa)
S355J2G1W	0	379	0	200
	0	360	0	192
	0	352	0	178
	0	319	0	130
	75	410	75	200
	75	350	75	163
	75	303	75	150
	75	265	75	132
	75	231	150	185
	150	365	150	182
	150	321	150	170
	150	277	150	160
	150	270	150	149
	225	385	225	185
	225	353	225	170
	225	325	225	160
	225	315	225	136
	225	274		
S355J0	0	400	0	224
	0	365	0	196
	0	275	0	184
	123	368	58	175
	100	298	50	151
	90	269	456	139
	82	247	139	139
	290	290	136	136
	275	275	133	133
	269	269		
	257	257		
	236	236		

**Table 3:** Parameters of regression equation.

Material	Stress Ratio R	Bending		Torsion	
		A	B	A	B
S355J2G1W	−1	−12.73	38.03	−14.83	39.28
	0	−17.29	30.78	−20.37	50.29
S355J0	−1	−7.19	23.93	−11.82	32.81
	0	−10.73	31.40	−25.25	59.76

**Table 4:** Parameters of the function ψ = f(N).

Material	Load Case	$\psi (N)\;=\;\eta \cdot N^{\lambda }$	
		η	λ
S355J2G1W	bending	1.01	−0.072
	torsion	0.82	−0.113
S355J0	bending	3.16	−0.164
	torsion	2.89	−0.131

## Data Availability

The data presented in this study are available on request from the corresponding author.

## References

[1] Goodman J. (1899). Mechanics Applied to Engineering.

[2] Gerber W. (1874). Bestimmung der zulossigne Spannungen in eisen Constructionen. Z. Bayer. Arch. Ing. Ver..

[3] Marin J. (1949). Biaxial tensin-torsion fatigue strengths of metals. J. Appl. Mech..

[4] Marin J. (1956). Interpretation of fatigue strengths for combined stresses. Proceedings of the International Conference on Fatigue of Metals.

[5] Kliman V. (1993). Prediction of Random Load Fatigue Life Distribution.

[6] Morrow J. (1968). In Fatigue design handbook, advances in engineering, Society of Automotive Engineers. Warrendale.

[7] Smith K., Watson P., Topper T. (1970). A stress strain function for the fatigue of metals. J. Mater..

[8] Manson S., Halford G. (2006). Fatigue and Durability of Structural Materials.

[9] Basquin O. (1910). The exponential law of endurance tests. Proc. Am. Soc. Test. Mater..

[10] Walker K. (1970). The Effect of Stress Ratio during Crack Propagation and Fatigue for 2024-T3 and 7075-T6 Aluminum. Effects of Environment and Complex Load History on Fatigue Life—ASTM STP 462.

[11] Łagoda T., Macha E., Pawliczek R. (2001). The influence of mean stress on fatigue life of 10HNAP steel under random loading. Int. J. Fatigue.

[12] Pawliczek R., Kluger K. (2013). Influence of irregularity coefficient of loading on calculated fatigue life. J. Theor. Appl. Mech..

[13] Gasiak G., Pawliczek R. (2003). Application of an energetic model for fatigue life prediction of the construction steels under bending, torsion and synchronous bending and torsion. Int. J. Fatigue.

[14] Kluger K., Karolczuk A., Derda S. (2020). Application of life-dependent material parameters to fatigue life prediction under multiaxial and non-zero mean loading. Materials.

[15] Kluger K., Pawliczek R. (2019). Assessment of Validity of Selected Criteria of Fatigue Life Prediction. Materials.

[16] Kluger K., Lagoda T. (2014). New energy model for fatigue life determination under multiaxial loading with different mean values. Int. J. Fatigue.

[17] Bohm M., Kluger K., Pochwala S., Kupina M. (2020). Application of the S-N curve mean stress correction model in terms of fatigue life estimation for random torsional loading for selected aluminum alloys. Materials.

[18] Pawliczek R., Lagoda T. (2021). Investigation of changes in fatigue damage caused by mean load under block loading conditions. Materials.

[19] Dowling N. (2009). Mean stress effects in strain–life fatigue. Fatigue Fract. Eng. Mater. Struct..

[20] Dowling N., Calhoun C., Arcari A. (2009). Mean stress effects in stress-life fatigue and the Walker equation. Fatigue Fract. Eng. Mater. Struct..

[21] Ince A., Glinka G. (2011). A modification of Morrow and Smith–Watson–Topper mean stress correction models. Fatigue Fract. Eng. Mater. Struct..

[22] Kwofie S., Chandler H.D. (2001). Low cycle fatigue under tensile mean stresses where cyclic life extension occurs. Int. J. Fatigue.

[23] Mrozinski S., Lipski A. (2012). Method for processing of the results of low-cycle fatigue tests. Mater. Sci..

[24] Mrozinski S., Piotrowski M. (2016). Effect of strain level on cyclic properties of S355 steel. Fatigue Failure and Fracture Mechanics. AIP Conf. Proc..

[25] Ligaj B., Szala G. (2010). Experimental verification of two-parametric models of fatigue characteristics by using the tests of S55J0 steel as an example. Pol. Marit. Res..

[26] Ligaj B., Soltysiak R. (2016). Problems of equivalent load amplitude in fatigue life calculations. Pol. Marit. Res..

[27] Pawliczek R., Rozumek D. (2020). The effect of mean load for S355J0 steel with increased strength. Metals.

[28] Lebedev A. Equivalent stress calculation for biaxial/multiaxial fatigue and fracture (experiment and theory). Proceedings of the 5th International Conference on Biaxial/Multiaxial Fatigue & Fracture.

[29] Pawliczek R., Rozumek D. (2020). Cyclic tests of smooth and notched specimens subjected to bending and torsion taking into account the effect of mean stress. Materials.

[30] Achtelik H., Kurek M., Kurek A., Kluger K., Pawliczek R., Łagoda T. (2018). Non-standard fatigue stands for material testing under bending and torsion loadings. AIP Conf. Proc..

[31] Woodard R. (2000). Interpolation of Spatial Data: Some Theory for Kriging. Technometrics.

